# Clinical features and outcomes of patients with wheat-dependent exercise-induced anaphylaxis: a retrospective study

**DOI:** 10.1186/s13223-022-00702-1

**Published:** 2022-07-05

**Authors:** Zhirong Du, Xiang Gao, Junda Li, Lun Li, Juan Liu, Jia Yin

**Affiliations:** 1 Department of Allergy, Peking Union Medical College Hospital, Chinese Academy of Medical Sciences and Peking Union Medical College, No. 1 Shuaifuyuan Street, Wangfujing, Beijing, 100730 China; 2 Beijing Key Laboratory of Precision Medicine for Diagnosis and Treatment of Allergic Disease, Beijing, 100730 China; 3 National Clinical Research Center for Dermatologic and Immunologic Diseases, Beijing, 100730 China

**Keywords:** Wheat-dependent exercise-induced anaphylaxis, Diagnosis, Management, Outcome

## Abstract

**Background:**

Wheat-dependent exercise-induced anaphylaxis (WDEIA) is a serious and potentially life-threatening form of wheat allergy. Further episodes can only be prevented by avoiding wheat ingestion or avoiding exercise after wheat intake. Anaphylaxis may recur in some patients post-diagnosis. This study aimed to analyze the clinical features and management/outcomes of WDEIA in China.

**Methods:**

We retrospectively analyzed the clinical characteristics, and laboratory testing of 197 patients with WDEIA. After diagnosis, the patients were followed up as outpatients to evaluate dietary/exercise choice and clinical outcomes.

**Results:**

Among the 197 WDEIA patients (median age, 37 years), 53.8% were male and 28.4% had other allergic disorders. The median duration of anaphylaxis before diagnosis was 16 months. Significant delays in diagnosis (> 1 years) were recorded in 52.7% of the patients, which has not decreased by years (*P* = 0.064). Exercise (83.8%), alcohol (12.2%), and nonsteroidal anti-inflammatory drugs (7.1%) were the most common cofactors. The most common clinical features were urticaria (100%), loss of consciousness (82.7%), dyspnea (50.8%), and hypotension (47.2%). Of the 197 eligible patients, 155 responded (78.7%), and 124 (80.0%) of which had no anaphylaxis post-diagnosis. A wheat-free diet prevented future anaphylaxis in 91.7% of the patients, followed by the avoidance of wheat combined with exercise (87%) and reduced wheat intake combined with exercise avoidance (80.5%).

**Conclusion:**

The diagnosis of WDEIA is frequently delayed. Therefore, when patients present with unexplained anaphylaxis, the possibility of WDEIA should be considered. A wheat-free diet or avoiding wheat combined with exercise or reduced wheat combined with exercise avoidance helps to significantly reduce the onset of future anaphylaxis. However, approximately one-fifth of patients continue to experience anaphylaxis post-diagnosis. Thus, these patients must always carry epinephrine autoinjectors.

**Supplementary Information:**

The online version contains supplementary material available at 10.1186/s13223-022-00702-1.

## Background

Wheat is a staple grain consumed by people worldwide, although adverse immunologic responses to wheat can trigger allergic reactions [1]. The food-challenge-defined prevalence of wheat allergy was 0.1% in Europe [2]. Recent data from the European Anaphylaxis Registry recognized wheat as the most common food elicitor of anaphylaxis in adults [3]. Depending on the route of exposure, wheat ingestion or inhalation can cause various IgE-mediated allergy disorders, including classic food allergy, baker’s asthma, rhinitis, and wheat-dependent exercise-induced anaphylaxis (WDEIA) [1]. WDEIA is a rare but life-threatening type of wheat allergy, manifesting as urticaria, angioedema, dyspnea, hypotension, collapse, and anaphylactic shock [4, 5]. It occurs after wheat consumption combined with cofactors, such as exercise, alcohol, aspirin/nonsteroidal anti-inflammatory drugs (NSAIDs), fatigue, and stress [6].

Omega-5 gliadin (ω-5 gliadin) and high‑molecular‑weight glutenin subunits have been considered the major allergens in cases of WDEIA [4, 5], and specific IgE to ω-5 gliadin and gluten have been indicated as the major tests to facilitate the diagnosis of WDEIA [8–10]. However, tests for these are not routinely available in clinical practice, and reports on the delayed time of diagnosis of WDEIA in China are insufficient. Currently, no standardized management strategies for patients with WDEIA exist [11]. In a short-term follow-up study [12, 13], anaphylaxis was successfully prevented in practically all WDEIA patients by avoiding wheat in association with other cofactors or a wheat-free diet. However, a multicenter evaluation in UK [11] has identified that 33% of WDEIA patients who avoided wheat in combination with exercise, and 71% of those who selected a gluten-free diet reported allergic reactions post-diagnosis. Meanwhile, data on the long-term control status of patients with WDEIA are insufficient in China.

Therefore, we aimed to analyze the clinical features and management/outcomes of WDEIA by presenting the detailed characteristics and follow-up data of a large cohort of patients with WDEIA in China.

## Methods

### Study population

Data of patients who were diagnosed with WDEIA in the Department of Allergy, Peking Union Medical College Hospital between May 2008 and April 2021 were collected. The diagnosis of WDEIA was made by an allergist who reviewed a clinical history compatible with anaphylaxis after wheat consumption and a positive specific IgE to omega-5 gliadin or gluten (> 0.35 kU/L) [11]. According to the World Allergy Organization Grading System for systemic allergic reactions, grades 4 and 5 are defined as anaphylaxis [14]. Anaphylaxis usually occurred when cofactors existed within 6 h of wheat ingestion. But there are no identifiable cofactors in certain cases [11, 15]. Exercise, NSAIDs, alcohol, stress, fatigue, and menstruation were considered as potential cofactors [6, 16]. Anaphylaxis caused by other causes, including food other than wheat, mastocytosis, medications, exposure to latex or insect stings, were excluded by detailed medical history collection and laboratory testing.

### Study design

This was a retrospective and descriptive study of patients who were diagnosed with WDEIA at our institution between 2008 and 2021. Patient data on sex, age, residential address, delay in diagnosis, clinical features, and laboratory examinations were extracted from medical records. To obtain the data on dietary choice and clinical outcomes, these patients were followed up by telephone, with follow-up data available for 155 cases.

### Laboratory studies

Baseline serum tryptase and specific IgE antibodies to wheat, gluten, ω-5 gliadin, rye, barley, and oat were measured using the ImmunoCAP system (Phadia AB, Uppsala, Sweden). Positive results of specific IgE were defined as > 0.35 kU/L.

### Statistical analysis

SPSS 25.0 (SPSS Inc, Chicago, IL, USA) was used for statistical analysis. A description analysis was performed to characterize the study population. Continuous variables were expressed as median and were compared using the Wilcoxon–Mann–Whitney or Kruskal–Wallis test. Categorical variables were expressed as percentage and were compared by Fisher test. *P* < 0.05 was considered significant.

## Results

### Demographic characteristics

Altogether, data of 197 patients diagnosed with WDEIA were analyzed, and follow-up data were available for 155 cases. The median age of the 197 patients was 37 years, ranging from 12 to 70 years. Of the patients, 46.2% (91/197) were women, and 53.8% (106/197) were men. The median age of men at the time of diagnosis was the same as that of women (38.5 years vs 35.0 years, *P* = 0.119). Additionally, 170 (86.3%) patients were from North China, and the remaining 27 (13.7%) patients were from South China.

### Time between initial anaphylaxis to the diagnosis of WDEIA has not changed by years

**Fig. 1:**
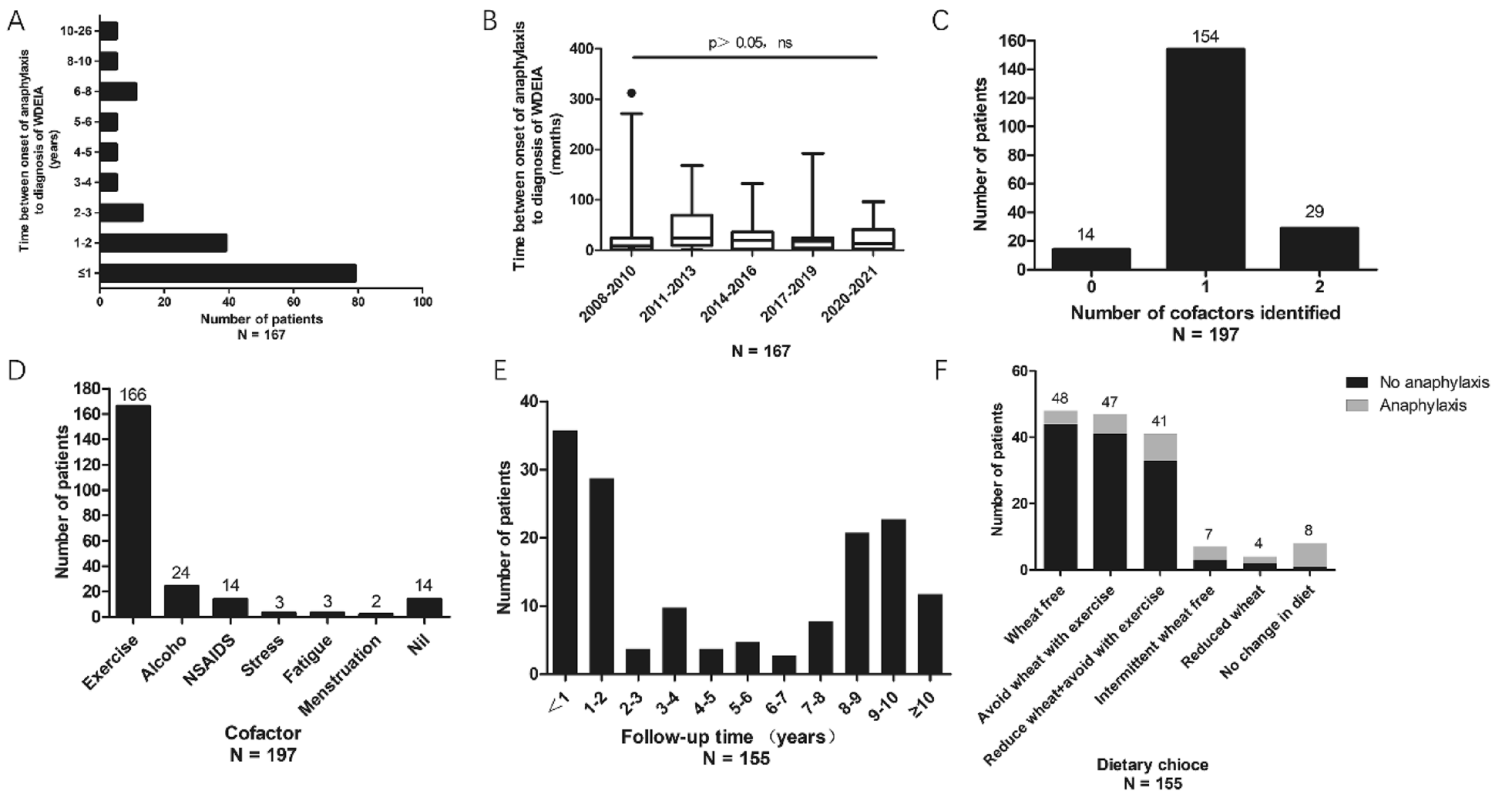
Characterization of patients with WDEIA. **A** Interval between onset of anaphylaxis and diagnosis of WDEIA. **B** Interval between onset of anaphylaxis and diagnosis of WDEIA in different years. **C** Cofactors identified during anaphylaxis. **D** Types of cofactors. **E** Follow-up time post diagnosis of WDEIA. **F** Dietary styles selected by patients post-diagnosis. *WDEIA*: wheat-dependent exercise induced anaphylaxis. *N*, total number of patients for each graph.

The median age of the first onset of anaphylaxis was 35 (12–64) years. The median duration between initial anaphylaxis to the diagnosis of WDEIA was 16 (0–312) months. Of the 167 patients for whom data were available, 47.3% were diagnosed within 1 year after the onset of anaphylaxis, and 52.7% experienced significant delays in diagnosis of > 1 year. The distribution of interval between the first onset of anaphylaxis and diagnosis is illustrated in Fig. 1A. However, the delay time of diagnosis has not decreased by years (*P* = 0.064) (Fig. 1B).

### Clinical features

The median frequency of anaphylaxis episodes was two, ranging from one to more than ten times. Regarding the frequency of anaphylaxis, 51 patients (25.9%) experienced anaphylaxis once, and 130 patients (66.0%) experienced it more than once before diagnosis, while the data from 16 (8.1%) patients were unavailable. Additionally, of the 197 patients, 78.2% reported one cofactor, 14.7% reported two cofactors, and 7.1% identified no cofactors (Fig. 1C). Exercise was the most common cofactor (84.3%), followed by alcohol (12.2%), and NSAIDs (7.1%). (Fig. 1D). Wheat ingestion preceded exercise and other cofactors. Of the 184 (93.4%) patients with available data, the duration between wheat intake and anaphylaxis were < 30 min in 94 (51.1%) patients, 30–60 min in 60 (32.6%) patients, and ≥ 1 h in 30 (16.3%) patients.

The clinical characteristics of the 197 patients varied. All patients had dermatologic allergic reactions. Meanwhile, 52.8%, 34.0%, and 93.4% reported respiratory, gastrointestinal, and cardiovascular symptoms, respectively. The most common clinical features were urticaria (100%), loss of consciousness (82.7%), dyspnea (50.8%), hypotension (47.2%), and blurred vision (39.1%). Details on clinical characteristics are listed in Table 1.

**Table 1:** Demographic and clinical characteristics of 197 patients with WDEIA

Variables	n (%)	Median (range)
Sex		
Male	106 (53.8)	
Female	91 (46.2)	
Residence		
North China	170 (86.3)	
South China	27 (13.7)	
Onset age (years)		35 (12–64)
Diagnosis age (years)		37 (12–70)
Number of anaphylactic episodes		2 (1– > 10)
Clinical features		
Dermatologic reactions	197 (100)	
Urticaria	197 (100)	
Angioedema	42 (21.3)	
Respiratory symptoms	104 (52.8)	
Dyspnea	100 (50.8)	
Throat angioedema	17 (8.6)	
Gastrointestinal symptoms	67 (34.0)	
Abdominal pain	26 (13.2)	
Diarrhea	7 (3.6)	
Nausea	23 (11.7)	
Vomiting	45 (22.8)	
Cardiovascular symptoms	184 (93.4)	
Blurred vision	77 (39.1)	
Dizzy	27 (13.7)	
Loss of consciousness	163 (82.7)	
Tachycardia	37 (18.8)	
Hypotension	93 (47.2)	
Others	75 (38.1)	
Incontinence	38 (19.3)	
Fatigue	34 (17.3)	
Convulsions	8 (4.1)	
Tinnitus	5 (2.5)	
Numbness	4 (2.0)	

A minority of patients had specific IgE testing to other cereals (rye, barley, and oat). Half of those tested had positive specific IgE to rye; and 9.1% and 9.2% of patients had positive specific IgE to barley and oat respectively (Table 2). Of the patients with WDEIA, 28.4% (56/197) had other allergic disorders, consisting of 21.8% (43) with allergic rhinitis, 2.5% (5) with allergic asthma, and 7.1% (14) with food allergy to other foods. The causative foods were seafood (n = 13), vegetables and fruits (n = 1), and nuts and seeds (n = 2). The median level of total IgE was 224 (16.1–2197) kU/L. The positive rate of specific IgE to wheat, gluten, and ω-5 gliadin was 52.7% (97/184), 86.5% (167/193), and 98.1% (152/155), respectively (Table 3). 40.6% (80/197) of patients had baseline serum tryptase measured. The median level of baseline tryptase was 3.17 (1.07–8.52) μg/L.

**Table 2:** Specific IgE testing for rye, barley, and oat

Blood test	sIgE to rye	sIgE to barley	sIgE to oat
Patients with data (n)	50	77	98
Positive IgE (> 0.35 kU/L)	25 (50%)	7 (9.1%)	9(9.2%)

*sIgE* specific immunoglobulin E

**Table 3:** Specific IgE testing for wheat, gluten, and ω-5 gliadin

Blood test	sIgE to wheat	sIgE to gluten	sIgE to ω-5 gliadin
Patients with data (n)	184	193	155
Positive IgE (> 0.35 kU/L)	97 (52.7%)	167 (86.5%)	152 (98.1%)
Positive result analysis			
Minimum	0.37	0.35	0.42
Maximum	63.50	31.20	49.40
Median	0.92	2.11	7.37

*sIgE* specific immunoglobulin E

### Follow-up and clinical outcomes

Follow-up data were available for 155 patients (78.7%). The median follow-up period was 46.7 (1.5–136.7) months. Of the 155 patients, 23.2% had follow-up for < 1 year, 30.3% had follow-up for 1 to 5 years, 38.8% had follow-up for 5 to 10 years, and 7.7% had follow-up for ≤ 10 years (Fig. 1E).

Of the 155 patients who were followed-up 124 (80.0%) had no anaphylaxis after diagnosis. A wheat-free diet (n = 48) was the commonest management strategy selected by the patients, followed by avoiding wheat in combination with exercise (n = 47), and reducing wheat consumption combined with exercise avoidance (n = 41); the incidence of anaphylaxis post-diagnosis among them was 8.3%, 12.8%, and 19.5%, respectively. Moreover, seven patients selected intermittent wheat free diet, and four reduced wheat ingestion, and 57.1% and 50.0% of them experienced anaphylaxis post-diagnosis, respectively. Eight patients did not change their dietary style, and anaphylaxis recurred in seven patients (87.5%) (Fig. 1F). The outcomes on future anaphylaxis were significantly different between the different dietary groups (*P* < 0.001). A wheat-free diet prevented future anaphylaxis successfully in 91.7% of patients, followed by the avoidance of wheat combined with exercise (87.2%) and reduced wheat combined with exercise avoidance (80.5%). The clinical characteristics of patients who had anaphylaxis following diagnosis has been shown in Additional file 1: Table S1, and other causes of allergic reactions were excluded. In the group with reduced wheat consumption combined with exercise avoidance, the patients with recurrent anaphylaxis were younger than those who had no anaphylaxis post-diagnosis (25.5 years vs 36.0 years, *P* = 0.04) (Additional file 1: Table S2).

Only one patient reported achieving wheat tolerance. This patient chose to avoid exercising after wheat ingestion for 5 years after diagnosis. On one incident, he ran quickly to catch a train 30 min after eating steamed stuffed buns. To his surprise, he had no allergic reactions, and from then on, he was glad to find that he could tolerate ingesting wheat and exercising soon after in the following 5 years. Unfortunately, wheat challenge examination was not allowed for ethical reasons, so evaluating the changes in the threshold of these patients was difficult.

## Discussion

As the most widely consumed food grain in China, wheat is reported as the commonest allergen of food-dependent exercise-induced anaphylaxis [12]. Unfortunately, WDEIA is a life-threatening disorder with no cure. To prevent future anaphylaxis, a wheat-free diet or the avoidance of wheat ingestion combined with cofactors is recommended [4, 15]. However, we have observed that after diagnosis, the dietary/lifestyle patterns selected by patients with WDEIA vary greatly, and some patients experienced recurrent anaphylaxis.

Similar with the population in previous studies [10, 11, 17], 54%–60% of the patients with WDEIA were men, and the median age of diagnosis was 41–44 years. Moreover, the median age of men at the time of diagnosis has been reported to be more than that of women [11], although the difference was not significant (38.5 years vs 35.0 years, *P* = 0.119) in our study. The relationship between the time of disease diagnosis and sex requires further analysis. In this study, the proportion of patients was higher from North China than from South China, which may be related to the different dietary habits of different regions.

Diagnosing WDEIA has long been challenging [18]. Our research revealed that the median duration of anaphylaxis was 16 months before diagnosis, and 52.7% of the patients experienced significant delays in diagnosis of > 1 year. The delay time of diagnosis was different between studies. In a study by Wong et al. [19], the average time taken for WDEIA to be diagnosed was 28.5 (2–62) months. The delay was 32 to 62 months before diagnosis in half of the patients. Meanwhile, in Kennard et al.’s [11] retrospective study of 132 patients with WDEIA in four UK centers, 66.7% of patients had a delay in diagnosis of > 1 year, including 40% of patients with a delay in diagnosis of 1 to 5 years and 29% with a delay in diagnosis of > 5 years. The time of misdiagnosis in our study may be shorter than that in previous studies [20], although we did not find a decreasing trend in the past 13 years. This may be because most patients had taken wheat as a staple grain for a long time without allergic symptoms, and they may not experience anaphylaxis after each wheat ingestion, leading to challenges in identifying wheat as the responsible allergen by both the physicians and patients.

In line with a study done by Kraft et al. [3], our study demonstrated that cardiovascular symptoms presented frequently and respiratory symptoms presented less commonly in anaphylaxis to wheat. Cofactors play important roles in anaphylaxis reactions in patients with WDEIA [21]. Exercise has been reported as a cofactor in 82.8% of reactions to wheat in adults [3], which is consistent with our data. Alcohol has been reported as a cofactor in 25% ofpatients [11], which is higher than that in our study. This may be related to different dietary habits. Exercise, NSAIDs, and alcohol have been reported to increase gastrointestinal permeability, thus inducing absorption of allergens from the gastrointestinal tract into the circulation [22–24]. Additionally, exercise may increase immunogenicity of ω-5 gliadin, and NSAIDs may affect mast cell degranulation [21, 25]. Similarly, Kennard et al. [11] reported that 11% of patients with WDEIA had no identifiable cofactor and it has been shown that cofactors may not be necessary if wheat intake is high enough [18], indicating that cofactors mainly reduce the threshold of immune response to wheat, which provides a possible explanation for why cofactors are not always identified. In addition, the definition of exercise varies widely among individual patients, which is difficult to evaluate. In this study, the positive rate of specific IgE to wheat, gluten, and ω-5 gliadin was 52.7%, 86.5%, and 98.1%, respectively. Similarly, Kennard et al. ^11^ have reported that in 132 patients, 59%, 76%, and 100%, were positive for specific IgE to wheat, gluten, and ω-5 gliadin, respectively. ω-5 gliadin and high molecular weight-gluten subunit (HMW-glutenin) are two main wheat allergens for WDEIA [9, 26]. The sensitivity of specific IgE antibodies to ω-5 gliadin and gluten was identified ^18^ to be both 100%, with specificities of 97% and 95%, respectively. Our previous study has identified that the sensitivity and specificity of combined specific IgE to gluten and omega-5 gliadin were 73.1% and 99%, respectively, which are valuable for the diagnosis of WDEIA [10]. We found that there is in vitro cross-reaction with rye, barley, or oat in a small number of patients with WDEIA, which has also been observed in another study [11]. Therefore, attention should be paid to potential cross-reactivity with other cereals. Herein, 80.0% of patients had no anaphylaxis post-diagnosis. A total wheat-free diet or the avoidance of wheat ingestion combined with cofactors is recommended to prevent further episodes of WDEIA [4, 15]. A wheat-free diet was the most effective management in this study. Meanwhile, in another study [11], a wheat-free diet led to only a 29% reduction in the risk of future anaphylaxis, and this may be due to different dietary/lifestyle habits between countries and patients. Similar to previous studies [12, 20], complete avoidance of wheat intake or avoidance of wheat products combined with exercise helped patients with WDEIA avoid further anaphylaxis effectively. Christensen et al. [27] have reported that the threshold in WDEIA may decrease in patients on a wheat-free diet, whereas the opposite is observed in patients with regular wheat intake. As a main staple, wheat can be difficult for patients to avoid. The avoidance of wheat in combination with exercise or reduced wheat ingestion combined with exercise avoidance could be selected, if considered safe for the patients. However, attention should be paid to the effect of unintentional exercise after wheat consumption.

Wheat tolerance can be achieved in 76% of children with wheat allergy within 18 years of age [28]. Meanwhile, studies have suggested that 20.5%, 54.2%, and 66.3% of children with a history of immediate-type allergic reaction to wheat, acquired tolerance to 200 g of udon noodles at 3, 5, and 6 years of age, respectively [29]. In a cohort study [30], 10 adult patients with wheat allergy were followed up for 5 years, and nine of them achieved wheat tolerance at the end of the follow-up, including two patients with wheat-dependent exercise-induced urticaria. However, there is a lack of long-term follow-up studies for patients with WDEIA. Among the 155 patients with WDEIA in our study, only one reported achieving wheat tolerance. Due to ethical reasons, evaluating the changes of wheat tolerance in these patients was impossible. The prognosis of WDEIA appears to be less favorable than that of wheat allergy. Further studies are needed to investigate the different mechanisms of allergen tolerance between WDEIA and wheat allergy.

As the first long-term follow-up study to investigate the management and outcomes of patients with WDEIA in China, this study provides reference for the treatment of WDEIA. However, this study had some limitations. First this study might have recall bias owing to its retrospective nature. Second, it was not possible to follow-up with all patients because the follow-up data of 155 patients were obtained by telephone, and 42 patients changed their telephone number or refused to answer the call. Thirdly, a provocation food challenge was not performed to confirm the diagnosis due to ethical concerns. Furthermore, wheat challenges are needed to determine changes in the threshold in WDEIA.

## Conclusions

In conclusion, our retrospective study found that the median duration between initial anaphylaxis to the diagnosis of WDEIA was 16 months, which indicates a delay of > 1 year in 52.7% of cases. The delay in diagnosis time has not decreased in years, which highlights the importance of improving the screening and identification of WDEIA in patients by clinicians. Exercise, alcohol, and NSAIDs were the commonest exacerbating cofactors. The commonest clinical features were urticaria, loss of consciousness, dyspnea, and hypotension. Only 80.0% of patients had no anaphylaxis post-diagnosis. Therefore, patients with WDEIA should continue to carry epinephrine autoinjectors throughout their life. Significant differences in the outcomes were observed between different diet choices. A wheat-free diet yielded the largest reductions in future anaphylaxis (91.7%), followed by the avoidance of wheat combined with exercise (87.2%) and reduced wheat combined with exercise avoidance (80.5%). Further studies are needed to investigate the changes in threshold of patients with WDEIA.

## Supplementary Information

**Additional file 1: Table S1.** The clinical characteristics of patients who had anaphylaxis following diagnosis. **Table S2.** Patient characteristics in the wheat-free diet, avoid wheat with exercise, and reduced wheat combined with exercise avoidance groups.

## Data Availability

Data of the participants are confidential. The datasets used and/or analyzed during the current study are available from the corresponding author on reasonable request.

## Abbreviations

WDEIA: Wheat-dependent exercise-induced anaphylaxis
NSAIDs: Nonsteroidal anti-inflammatory drugs
ω-5 gliadin: Omega-5 gliadin
