# Supplementary material for: Clinical features and outcomes of patients with wheat-dependent exercise-induced anaphylaxis: a retrospective study
Source: Allergy Asthma Clin Immunol. 2022 Jul 5;18:61. doi: 10.1186/s13223-022-00702-1 (PMC9254488; doi:10.1186/s13223-022-00702-1)
Supplement: Supplementary file 1 — Additional file 1: Table S1. The clinical characteristics of patients who had anaphylaxis following diagnosis. Table S2. Patient characteristics in the wheat-free diet, avoid wheat with exercise, and reduced wheat combined with exercise avoidance groups. [file 13223_2022_702_MOESM1_ESM.docx]

Supplementary table 1. The clinical characteristics of patients who had anaphylaxis following diagnosis

| Patient | Dietary choice | Types of wheat ingested | Wheat ingested accidentally or intentionally | Cofactors | Exercise accidentally or intentionally |
| --- | --- | --- | --- | --- | --- |
| 1 | Avoid wheat with exercise | Pancake | Intentionally | Walking at slow speed | Accidentally |
| 2 | No change in diet | Noodles | Intentionally | Walking at slow speed | Intentionally |
| 3 | Wheat free | Hoecake | Accidentally | Walking at daily speed | Intentionally |
| 4 | Avoid wheat with exercise | Dumplings | Intentionally | Walking at slow speed | Accidentally |
| 5 | No change in diet | Steamed bun | Intentionally | Walking at slow speed | Intentionally |
| 6 | Avoid wheat with exercise | Steamed bun | Intentionally | Walking at slow speed | Accidentally |
| 7 | Intermittent wheat free | Bread | Intentionally | Running | Intentionally |
| 8 | No change in diet | Steamed bun | Intentionally | Walking at slow speed | Intentionally |
| 9 | No change in diet | Moon cake | Intentionally | Climbing stairs | Intentionally |
| 10 | Intermittent wheat free | Steamed bun | Intentionally | Walking at slow speed | Intentionally |
| 11 | Avoid wheat with exercise | Noodles | Intentionally | Walking at daily speed | Accidentally |
| 12 | Avoid wheat with exercise | Steamed bun | Intentionally | Walking at slow speed | Accidentally |
| 13 | Intermittent wheat free | Pastry | Accidentally | Walking at slow speed | Intentionally |
| 14 | Reduced wheat | Steamed bun | Intentionally | Walking at slow speed | Intentionally |
| 15 | Wheat free | Biscuits | Accidentally | Running | Intentionally |
| 16 | Intermittent wheat free | Ice cream | Accidentally | Playing table tennis | Intentionally |
| 17 | No change in diet | Bread | Intentionally | Walking at daily speed | Intentionally |
| 18 | Wheat free | Mixed grain pancake | Accidentally | Walking at slow speed | Intentionally |
| 19 | No change in diet | Wonton | Intentionally | Walking at quick speed | Intentionally |
| 20 | Wheat free | Mixed grain pancake | Accidentally | Playing badminton | Intentionally |
| 21 | Reduce wheat + avoid with exercise | Steamed stuffed bun | Intentionally | Walking at slow speed | Accidentally |
| 22 | Reduce wheat + avoid with exercise | Noodles | Intentionally | Walking at daily speed | Accidentally |
| 23 | Reduce wheat + avoid with exercise | Naked oats noodle | Accidentally | Walking at daily speed | Accidentally |
| 24 | No change in diet | Noodles | Intentionally | Walking at slow speed | Intentionally |
| 25 | Avoid wheat with exercise | Steamed bun | Intentionally | Walking at daily speed | Accidentally |
| 26 | Reduce wheat + avoid with exercise | Steamed stuffed bun | Intentionally | Walking at daily speed | Accidentally |
| 27 | Reduce wheat + avoid with exercise | Clay oven rolls | Intentionally | Walking at slow speed | Accidentally |
| 28 | Reduce wheat + avoid with exercise | Dumplings | Intentionally | Walking at daily speed | Accidentally |
| 29 | Reduce wheat + avoid with exercise | Roast fish | Accidentally | Walking at daily speed | Intentionally |
| 30 | Reduced wheat | Fried shrimp cake | Accidentally | Walking at daily speed | Intentionally |
| 31 | Reduce wheat + avoid with exercise | Biscuits | Intentionally | Walking at quick speed | Accidentally |

Supplementary file 2. Patient characteristics in the wheat-free diet, avoid wheat with exercise, and reduced wheat combined with exercise avoidance groups

| Variables | Wheat-free diet (N=48) | | | Avoid wheat with exercise (N=47) | | | Reduced wheat+avoid with exercise (N=41) | | |
| --- | --- | --- | --- | --- | --- | --- | --- | --- | --- |
|  | Anaphylaxis | No Anaphylaxis | *P* | Anaphylaxis | No Anaphylaxis | *P* | Anaphylaxis | No Anaphylaxis | *P* |
| Male (%) | 0 | 52.27 | 0.11 | 66.67 | 58.53 | 1.00 | 50.00 | 66.67% | 0.43 |
| Age at first onset of anaphylaxis (years) | 35.00 | 38.00 | 0.52 | 36.50 | 36.00 | 0.99 | 25.50 | 36.00 | 0.04 |
| T-IgE (kU/L) | 387.80 | 237.00 | 0.69 | 276.00 | 221.00 | 0.38 | 227.00 | 179.00 | 0.48 |
| sIgE to wheat (kU/L) | 0.49 | 0.54 | 1.0 | 0.68 | 0.45 | 0.73 | 0.27 | 0.47 | 0.72 |
| sIgE to gluten (kU/L) | 4.11 | 2.25 | 0.58 | 1.16 | 1.30 | 0.73 | 1.58 | 1.41 | 1.00 |
| sIgE to ω-5 gliadin (kU/L) | 29.90 | 7.75 | 0.41 | 11.40 | 5.50 | 0.11 | 14.20 | 6.01 | 0.16 |

N, number; T-IgE, total immunoglobulin E; sIgE, specific immunoglobulin E

Categorical variables were expressed as percentage and compared by Fisher test; Continuous variables were expressed as median and compared by Wilcoxon Mann-Whitney
